# Effects of independent versus dependent stressful life events on major symptom domains of schizophrenia

**DOI:** 10.1038/s41537-023-00415-3

**Published:** 2023-12-08

**Authors:** Yizhou Ma, Joshua Chiappelli, Mark D. Kvarta, Heather Bruce, Andrew van der Vaart, Eric L. Goldwaser, Xiaoming Du, Hemalatha Sampath, Samantha Lightner, Jane Endres, Akram Yusuf, Alexa Yuen, Samantha Narvaez, Danny Campos-Saravia, Peter Kochunov, L. Elliot Hong

**Affiliations:** 1 Maryland Psychiatric Research Center, Department of Psychiatry, University of Maryland School of Medicine, Baltimore, MD USA; 2 Department of Psychiatry, Weill Cornell Medical College/New York-Presbyterian Hospital, New York, NY USA

**Keywords:** Schizophrenia, Human behaviour

## Abstract

We evaluated two models to link stressful life events (SLEs) with the psychopathology of schizophrenia spectrum disorders (SSD). We separated SLEs into independent (iSLEs, unlikely influenced by one’s behavior) and dependent (dSLEs, likely influenced by one’s behavior). Stress-diathesis and stress generation models were evaluated for the relationship between total, i- and d- SLEs and the severity of positive, negative, and depressive symptoms in participants with SSD. Participants with SSD (*n* = 286; 196 males; age = 37.5 ± 13.5 years) and community controls (*n* = 121; 83 males; 35.4 ± 13.9 years) completed self-report of lifetime negative total, i- and d- SLEs. Participants with SSD reported a significantly higher number of total SLEs compared to controls (*B* = 1.11, *p* = 6.4 × 10^–6^). Positive symptom severity was positively associated with the total number of SLEs (*β* = 0.20, *p* = 0.001). iSLEs (*β* = 0.11, *p* = 0.09) and dSLEs (*β* = 0.21, *p* = 0.0006) showed similar association with positive symptoms (*p* = 0.16) suggesting stress-diathesis effects. Negative symptom severity was negatively associated with the number of SLEs (*β* = –0.19, *p* = 0.003) and dSLEs (*β* = −0.20, *p* = 0.001) but not iSLEs (*β* = –0.04, *p* = 0.52), suggesting stress generation effects. Depressive symptom severity was positively associated with SLEs (*β* = 0.34, *p* = 1.0 × 10^–8^), and the association was not statistically stronger for dSLEs (*β* = 0.33, *p* = 2.7 × 10^–8^) than iSLEs (*β* = 0.21, *p* = 0.0006), *p* = 0.085, suggesting stress-diathesis effects. The SLE – symptom relationships in SSD may be attributed to stress generation or stress-diathesis, depending on symptom domain. Findings call for a domain-specific approach to clinical intervention for SLEs in SSD.

## Introduction

Stressful life events (SLEs) have been implicated in the etiology and symptomatology of schizophrenia spectrum disorders (SSD) since its initial conceptualization [1–4]. Two causal heuristics, stress-diathesis and stress generation, may explain the underlying relationship between SLEs and SSD. The stress-diathesis model posits that SLEs may act as an environmental risk factor for and lead to the onset and exacerbation of SSD in individuals with genetic predisposition to the illness [5]. This model is supported by observations that SLEs often precede the onset and relapse of psychosis in patients [6–9]. The stress generation hypothesis [10, 11] suggests that individuals with SSD may experience a higher number of SLEs because of SSD-driven behavior. This hypothesis has been mainly tested in major depressive disorder and partly accounts for the relationship between SLEs and the severity of depressive symptoms [12–14]. Individuals with SSD experience symptoms across multiple clinical domains and stress-diathesis and stress generation hypotheses may apply differently to each domain, leading to different implications for prevention and intervention strategies.

**Fig. 1:**
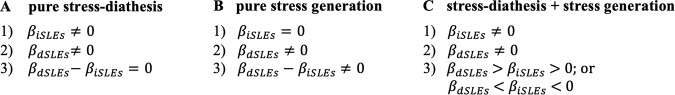
Heuristics used to evaluate stress-diathesis and stress generation models based on coefficient patterns. **A** pure stress-diathesis and no stress generation effects. **B** pure stress generation and no stress-diathesis effects. **C** a combination of stress-diathesis and stress generation effects. β standardized coefficients, SLEs stressful life events, iSLEs independent SLEs, dSLEs dependent SLEs.

We propose to evaluate stress-diathesis and stress generation models by separating SLEs into independent and dependent life events based on the heuristics summarized in Fig. 1 [11, 15]. “Independent” SLEs (iSLEs) are defined as events that are considered independent of one’s behavior, such as experiencing early life adversity and deaths in family. “Dependent” SLEs (dSLEs) are defined as events that are influenced by one’s behavior, including experiencing legal issues due to substance use, job loss due to impaired occupational functioning, and interpersonal conflicts. The stress-diathesis model does not predict differences in the associations with symptom severity between iSLEs and dSLEs (Fig. 1A). Stress generation posits that dSLEs, but not iSLEs will be associated with symptom severity because only dSLEs are among the consequences of experiencing the symptoms (Fig. 1B). When stress-diathesis and stress generation both contribute to the relationship between SLEs and symptoms, relationship with symptoms would be present for iSLEs and stronger for dSLEs (Fig. 1C). For example, assuming stress-diathesis and stress generation mechanisms, both iSLEs and dSLEs can lead to increases in depressive symptoms (stress-diathesis). However, increases in depressive symptoms can only lead to further dSLEs (stress generation) but not iSLEs because the latter are independent from one’s behavior. In other words, depressive symptoms can only form a positive feedback loop with dSLEs but not iSLEs. To the best of our knowledge, this is the first systematic effort to apply this approach to studying the SLE – symptom relationship in SSD.

Patients with SSD report positive, negative, and affective symptoms [16–18]. Positive symptoms include presence of abnormal thoughts, perceptions, or behavior such as delusions, hallucinations, and disorganization. Negative symptoms are deficits of normal functioning and include avolition, anhedonia, alogia, and others. Affective symptoms include mainly depression. The literature suggests that the relationship between SLEs and symptom severity is not uniform across symptom domains in SSD. The stress-diathesis model has been used to explain the association between positive symptoms and SLEs [19–21]. The relationship between SLEs and symptoms from other domains is more complex. Several studies did not observe a significant relationship between negative symptoms and SLEs [19, 22, 23]. However, a longitudinal study supported both stress-diathesis and stress generation effects [20]. Moreover, the longitudinally derived associations were negative and higher SLEs predicted fewer negative symptoms and vice-versa. As this study evaluated the total number of SLEs and did not distinguish between iSLEs and dSLEs, the observed bidirectional relationship was challenging to interpret. Higher negative symptoms may be associated with reduced social activities and fewer dSLEs and therefore separating iSLEs and dSLEs may help to reconcile this divergence. The relationship between SLEs and depressive symptoms in SSD [20, 22] paralleled the stress-diathesis but not stress-generation findings reported in major depressive disorder [15].

In this study, we examined the relationships between total, i- and d- SLEs and the severity of positive, negative, and depressive symptoms in SSD. We hypothesized that both iSLEs and dSLEs would be positively associated with positive symptom severity, supporting stress-diathesis but not stress-generation models (Fig. 1A). We hypothesized that dSLEs, but not iSLEs would be negatively associated with negative symptom severity, suggesting that previously reported negative relationship between SLEs and negative symptoms reflected a stress generation effect (Fig. 1B). We predicted that depressive symptoms would be positively associated with iSLEs and dSLEs, and the relationship would be stronger for the latter, reflecting stress generation in addition to stress-diathesis effects (Fig. 1C).

To further establish and clarify the relationship between SLEs and symptoms of SSD should ultimately facilitate more effective prevention and intervention strategies. Conceptualizing certain symptoms of SSD as responses to SLEs may facilitate the integration of ongoing stress assessment, trauma-informed care, and stress management in SSD treatment. Providers and researchers may draw parallels between SSD and stress-related disorders and borrow from these fields. On the other hand, understanding that some symptoms of SSD may alter patients’ likelihood of experiencing SLEs may facilitate the transition from a symptom-reduction to a recovery-oriented approach, where patients and their treatment teams work to diminish the impact of symptoms on real-life experiences.

## Methods

### Participants

Participants with schizophrenia spectrum disorders (SSD, *n* = 286; 196 males; 37.5 ± 13.5 years) and community controls (*n* = 121; 83 males; 35.4 ± 13.9 years) completed self-report on major negative stressful life events (SLEs) at the Maryland Psychiatric Research Center (MPRC) from 2010 to 2022 (Table 1). The community controls were drawn from a larger pool with stratified random sampling to match participants with SSD on race by sex composition. Participants with SSD were recruited from outpatient clinics at the MPRC and neighboring mental health outpatient clinics. Community controls were recruited through media advertisements from the Greater Baltimore area. Inclusion criteria for participants with SSD were a diagnosis of schizophrenia or schizoaffective disorder based on the Structured Clinical Interview for DSM-IV or -5 (SCID-IV or -5) at the time of recruitment. Inclusion criteria for community controls included no family history of psychosis in the prior two generations and no current diagnosis of severe mental illness based on SCID, although history of previous single episode depression was allowed. Exclusion criteria for all participants included current or past major medical and neurological illnesses, history of head injury with cognitive sequelae, intellectual disability, current substance abuse or substance dependence within the past 6 months based on SCID, and positive urine toxicology screening. Study protocols were approved by the University of Maryland Institutional Review Board. Participants provided written informed consent before participation. The authors assert that all procedures contributing to this work comply with the ethical standards of the relevant national and institutional committees on human experimentation and with the Helsinki Declaration of 1975, as revised in 2008.

**Table 1:** Sample characteristics.

	Schizophrenia spectrum disorders			Community controls			Statistics
	*n* (%)	Mean (sd)	Range	*n* (%)	Mean (sd)	Rrange	*t* or χ^2^
*N*	286	–	–	121	–	–	–
Age (years)	–	37.5 (13.5)	17.9–66.3	–	36.8 (13.9)	18.9–62.8	n.s.
Sex							
Male	196 (68.5)	–	–	83 (68.6)	–	–	n.s.
Female	90 (31.5)	–	–	38 (31.4)	–	–	
Race							
Black	139 (48.6)	–	–	59 (48.8)	–	–	n.s.
White	127 (44.4)	–	–	54 (44.6)	–	–	
Asian	7 (2.4)	–	–	5 (4.1)	–	–	
American Indian	2 (0.7)	–	–	0 (0.0)	–	–	
Mixed^a^	4 (1.4)	–	–	1 (0.8)	–	–	
unspecified	7 (2.4)	–	–	2 (1.7)	–	–	
SLEs							
Total SLEs	–	4.8 (2.4)	0–10	–	3.7 (2.0)	0–8	*t*(405) = 4.57^***^
iSLEs	–	1.6 (1.2)	0–5	–	1.5 (1.1)	0–5	n.s.
dSLEs	–	2.4 (1.3)	0–5	–	1.7 (1.2)	0–5	*t*(391) = 4.62^***^
Clinical Symptoms							
negative	–	26.2 (16.1)	0–59	–	–	–	–
positive	–	14.2 (6.0)	7–32	–	–	–	–
trait depression	–	19.6 (16.2)	0–72	–	8.9 (8.9)	0–40	*t*(383) = 6.61^***^
state depression	–	15.2 (14.1)	0–60	–	5.8 (7.5)	0–37	*t*(385) = 6.86^***^
Medication							
Typical	35 (12.2)	–	–	–	–	–	–
Atypical	187 (65.4)	–	– –	–	–	–	–
Typical + Atypical	27 (9.4)	–	–	–	–	–	–
No antipsychotics	24 (8.4)	–	–	–	–	–	–
unreported	13 (4.5)	–	–	–	–	–	–
CPZ equivalents	–	507.6 (632.6)	0–5400	–	–	–	–

*sd* standard deviation, *SLEs* stressful life events, *iSLEs* independent SLEs, *dSLEs* dependent SLEs, *CPZ* chlorpromazine, *n.s.* not significant.

^a^In participants with schizophrenia spectrum disorder: 2 Black and American Indian, 1 Asian and Hawaiian, and 1 White and other. In community controls: 2 Black and Asian, 1 Black and White, 1 Black and other, 1 Black, White, and other, 1 White and unknown race, 1 American Indian and other. *p < 0.05, **p < 0.01, ***p < 0.001.

### Stressful life events

Participants completed a self-report questionnaire on lifetime major negative stressful life events (SLEs). Ten event types were surveyed: (1) death of a spouse, father, mother, child, sibling, or significant other; (2) suffered from a serious illness, serious injury, or traffic accident; 3) a significant other suffered from a serious illness or a serious injury; (4) divorce or break-up of a relationship (you or your parents); (5) unusual, extremely stressful work or school; (6) experienced violence, a sexual assault, or a robbery; (7) lost a primary job, or substantial financial loss (you or your parent); 8) experienced a legal dispute or disputes; (9) had a hospitalization due to mental illness or substance use problems; and (10) other serious life event not listed above. For each event, participants indicated whether they have never experienced the event, experienced it over 10 years ago, within 1–10 years, within 1 year, or within the last 6 months. They could also write down their age at the time of the event. We defined total SLEs as the number of SLE types that a participant endorsed as ever experienced.

#### Independent and dependent SLEs

The distinction between independent and dependent life events is ideally made through interview [15, 24]. However, categorization has also been done based on convention or raters’ consensus and with self-report data [23, 25–34]. For example, serious injury, illness, or death of others is typically considered independent [26–28, 30, 31, 34]. Another example of independent life events is parental divorce [28]. On the other hand, events involving interpersonal difficulties, such as divorce, are typically considered dependent as one’s condition may directly or indirectly contribute to such events [15, 27, 28, 34]. Another common category of dependent events is those potentially under one’s control or result from one’s behavior, such as school and job difficulties and legal problems [26–28, 34].

Based on convention in the literature, we categorized likely independent SLEs (iSLEs) and potentially dependent SLEs (dSLEs) as follows. Events categorized as likely iSLEs included: (1) death of a spouse, father, mother, child, sibling, or significant other; (2) a significant other suffered from a serious illness or a serious injury; (3) divorce or break-up of a relationship (you or your parents) when happened before age 18; (4) experienced violence, a sexual assault, or a robbery; and (5) lost a primary job, or substantial financial loss (you or your parent) when happened before age 18. Events categorized as likely dSLEs included: (1) divorce or break-up of a relationship (you or your parents) when happened at or after age 18; (2) unusual, extremely stressful work or school; (3) lost a primary job, or substantial financial loss (you or your parent) when happened at or after age 18; (4) experienced a legal dispute or disputes; and (5) had a hospitalization due to mental illness or substance use problems. One event, “suffered from a serious illness, serious injury, or traffic accident”, was deemed uncategorizable without more details and was excluded from the categorization. Total iSLEs or dSLEs was the number of likely iSLE or dSLE types that a participant endorsed as ever experienced.

Somewhat arbitrarily, we used age 18 years old to separate “divorce or break-up of a relationship”: events that took place when participant was a minor (before 18) were assumed to be parental divorce or break-up thus iSLEs, whereas events that happened when participant was an adult (at or after 18) dSLEs. The same applied to “lost a primary job, or substantial financial loss”. To avoid undue influence of this decision on our findings, we alternatively defined iSLEs and dSLEs which did not include these two types of events. These findings are presented in supplementary results.

### Clinical symptoms

In participants with SSD, we measured positive symptoms using the positive symptom subscale of the 20-item version of the Brief Psychiatric Rating Scale [35], which included items on grandiosity, conceptual disorganization, hallucinatory behavior, and unusual thought content [36]. We measured negative symptoms as the total score on the Brief Negative Symptoms Scale [37]. We measured depressive symptoms with the Maryland Trait and State Depression scale [38], which captures symptoms of depression within the past 7 days (state depression) and throughout their adult life (trait depression). An exploratory factor analysis confirmed that the latent structure of these symptoms was consistent with positive, negative, and depressive symptoms measured with their respective instrument (see supplementary results). Among the 286 participants with SSD, 256 had data for positive symptoms, 258 for negative symptoms, and 270 for depressive symptoms.

Because we measured lifetime SLEs rather than SLEs within a timeframe before the assessment, we focused on the relationship between SLEs and depressive symptoms throughout adulthood (i.e., trait depression). We reported results for state depression in supplementary materials. On the other hand, positive and negative symptom severity can be viewed as the sum of 1) the temporally stable component of symptoms, 2) the fluctuating component of symptoms, and 3) measurement error. The temporal stability of positive and negative symptom severity is supported by previous studies that followed patients with schizophrenia for up to two years [39–41]. Previous studies have reported that history of trauma and stressful life events predicted current symptom severity in psychosis [42–44].

### Medication

Among the 286 participants with SSD, 27 were on both typical and atypical antipsychotics, 35 on typical antipsychotics only, 187 on atypical antipsychotics only, 24 not on any antipsychotics, and 13 deemed to not have reliable medication information. Chlorpromazine (CPZ) equivalents were calculated in 218 (81.3%) patient participants who had usable medication and dosage information.

### Analysis

We first compared SLEs in participants with SSD and community controls with independent sample *t* or χ^2^ tests. Next, in participants with SSD, we examined the association between total SLEs and positive, negative, and depressive symptoms by regressing each symptom onto total SLEs and total SLEs × sex interaction in linear regression models when controlling for age. SLEs × sex interaction was removed if not significant and a covariate of sex was included instead. We then examined the respective association of total iSLEs and dSLEs with clinical symptoms using the same approach. To determine if there was a significant difference in the strengths of association between total iSLEs and dSLEs with the same symptom, we entered total iSLEs and dSLEs in the same model to explain the symptoms and tested if there was a significant difference in their standardized coefficients (H0: *β*_iSLEs_ – *β*_dSLEs_ = 0) using the linearHypothesis function in the car package (car-3.1–0) [45] in R. All analyses were performed with the R statistical package (R-4.2.0) [46]. Statistical models that additionally controlled for antipsychotic medication use were tested separately, due to around 20% of missing data for CPZ equivalents.

## Results

### SLEs in participants with SSD and community controls

Table 1 summarizes the sample characteristics. Participants with SSD reported 4.8 ± 2.4 total SLEs, 1.6 ± 1.2 total iSLEs, and 2.4 ± 1.3 total dSLEs. Community controls reported 3.7 ± 2.0 total SLEs, 1.5 ± 1.1 total iSLEs, and 1.7 ± 1.2 total dSLEs. Participants with SSD reported significantly more total SLEs than community controls (*B* = 1.11, 95% CI = [0.63, 1.59], t(405) = 4.57, *p* = 6.4 × 10^–6^), as well as significantly more dSLEs (*B* = 0.67, 95% CI = [0.38, 0.95], t(391) = 4.62, *p* = 5.3 × 10^–6^) but not iSLEs (*B* = 0.12, 95% CI = [–0.13, 0.37], t(391) = 0.93, *p* = 0.35). Given the significant group differences in total SLEs and dSLEs, similar iSLEs between the groups provided some support that the iSLE categorization may indeed capture largely independent events.

Supplementary Table 3 illustrates comparison by each event type. Participants with SSD were significantly more likely to report suffering from a serious illness, serious injury, or traffic accident**;** divorce or break-up of a relationship before 18 (presumably in parents); unusual, extremely stressful work or school; and hospitalization due to mental illness or substance use problem. On the other hand, community controls were significantly more likely to report divorce or break-up of a relationship at or after 18 (presumably in self). Prevalence of other types of SLEs was not significantly different.

### Relationships between SLEs and clinical symptoms in SSD

**Fig. 2:**
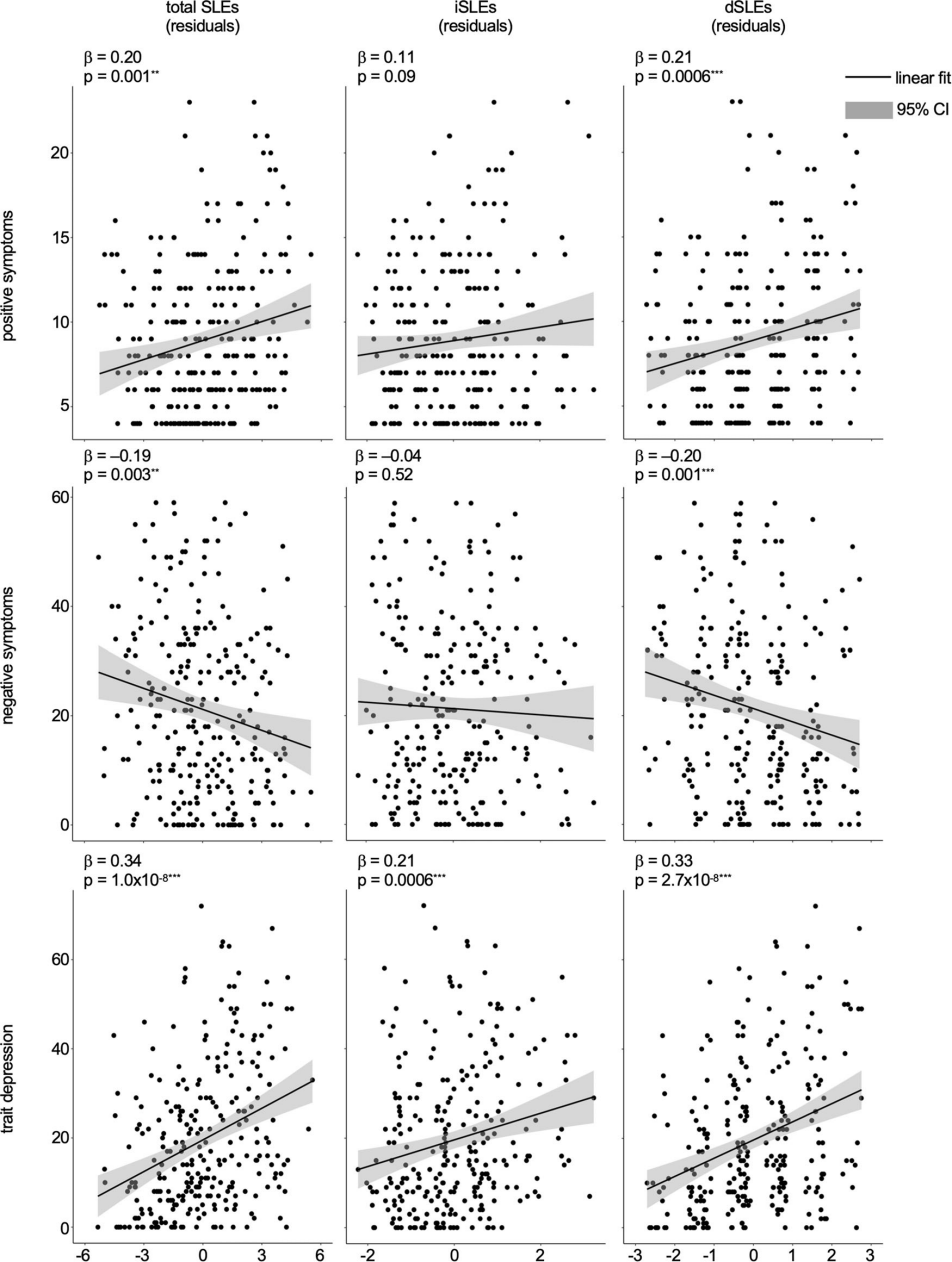
Association between total, independent, and dependent SLEs with clinical symptoms when controlling for age and sex. β standardized coefficients, SLEs stressful life events, iSLEs independent SLEs, dSLEs dependent SLEs. *p < 0.05, **p < 0.01, ^***^p < 0.001. All *p* values are two-tailed.

No significant SLEs × sex interaction was found for any symptom domain and SLEs × sex interaction was dropped from regression models. The relationships of SLEs with positive and negative symptoms as well as trait depression when adjusting for age and sex are shown in Table 2 and Fig. 2.

**Table 2:** Associations between SLEs and positive and negative symptoms as well as trait depression.

Symptoms	SLEs				iSLEs				dSLEs				*β*_iSLEs_ – *β*_dSLEs_
	β	*t*	df	*p* value	β	*t*	df	*p* value	β	*t*	df	*p* value	*p* value
Controlling for age and sex													
Positive	0.20	3.30	255	0.001^**^	0.11	1.70	249	0.09	0.21	3.47	249	0.0006^***^	0.16
Negative	−0.19	−2.97	254	0.003^**^	−0.04	−0.64	248	0.52	−0.20	−3.24	248	0.001^***^	0.039^*^
Trait depression	0.34	5.92	266	1.0 × 10^–8***^	0.21	3.47	260	0.0006^***^	0.33	5.74	260	2.7 × 10^–8***^	0.085
Controlling for age, sex, and CPZ equivalents													
Positive	0.14	2.05	197	0.042^*^	0.09	1.35	193	0.18	0.16	2.40	193	0.017^*^	0.40
Negative	−0.23	−3.16	197	0.002^**^	−0.06	−0.88	193	0.38	−0.22	−3.17	193	0.002^**^	0.070
Trait depression	0.32	4.55	202	9.2 × 10^–6***^	0.18	2.52	199	0.013^*^	0.33	4.73	199	4.2 × 10^–6***^	0.082

All *p* values are two-tailed.

*β* standardized coefficients, *SLEs* stressful life events, *iSLEs* independent SLEs, *dSLEs* dependent SLEs. *CPZ* chlorpromazine. *p < 0.05, **p < 0.01, ***p < 0.001.

Positive symptoms were significantly and positively associated with total SLEs (*β*_SLEs_ = 0.20, 95% CI = [0.08, 0.33], *t*(255) = 3.30, *p* = 0.001). When broken down by independence, positive symptoms were significantly associated with dSLEs (*β*_dSLEs_ = 0.21, 95% CI = [0.09, 0.33], *t*(249) = 3.47, *p* = 0.0006), but the association with iSLEs did not reach significance (*β*_iSLEs_ = 0.11, 95% CI = [–0.02, 0.23], *t*(249) = 1.70, *p* = 0.09). However, statistical tests suggested no significant difference between *β*_dSLEs_ and *β*_iSLEs_ (*p* = 0.16).

Negative symptoms were significantly and negatively associated with total SLEs (*β*_SLEs_ = –0.19, 95% CI = [–0.31, –0.06], *t*(254) = –2.97, *p*  = 0.003). This relationship held for dSLEs (*β*_dSLEs_ = –0.20, 95% CI = [–0.32, –0.08], *t*(248) = –3.24, *p* = 0.001) but not iSLEs (*β*_iSLEs_ = –0.04, 95% CI = [–0.17, –0.09], *t*(248) = –0.64, *p* = 0.52). Statistical tests showed that *β*_dSLEs_ was significantly different from *β*_iSLEs_ (*p* = 0.039).

Trait depression was significantly and positive associated with total SLEs (*β*_SLEs_ = 0.34, 95% CI = [0.23, 0.46], *t*(266) = 5.92, *p* = 1.0 × 10^–8^), as well as both iSLEs (*β*_iSLEs_ = 0.21, 95% CI = [0.09, 0.34], *t*(260) = 3.47, *p* = 0.0006) and dSLEs (*β*_dSLEs_ = 0.33, 95% CI = [0.22, 0.45], *t*(260) = 5.74, *p* = 2.7 × 10^–8^). The difference between *β*_dSLEs_ and *β*_iSLEs_ did not reach significance (*p* = 0.085).

In the 218 participants with available information, CPZ equivalents had a significant positive association with positive symptoms when controlling for age and sex (*β* = 0.13, 95% CI = [0.003, 0.27], t(198) = 2.02, *p* = 0.044). CPZ equivalents were not significantly associated with negative symptoms (*β* = 0.06), trait depression (*β* = –0.06), state depression (*β* = –0.02), SLEs (*β* = –0.06), iSLEs (*β* = –0.07), or dSLEs (*β* = –0.04), all p’s > 0.30. Additionally controlling for CPZ equivalent did not change the statistical significance of most findings (Table 2) except that the difference between *β*_dSLEs_ and *β*_iSLEs_ was no longer significant for negative symptoms. However, the estimated coefficients when controlling versus not controlling for CPZ equivalents were very similar, suggesting that results that became insignificant after controlling for CPZ equivalents were likely due to reduced statistical power (around 20% of missing data).

Using alternatively defined iSLEs and dSLEs (excluding events 4 and 7) yielded largely the same conclusions (Supplementary Table 4). One difference was that alternatively defined iSLEs were significantly positively associated with positive symptoms (*β*_iSLEs_ = 0.19, 95% CI = [0.07, 0.32], *t*(255) = 2.97, *p* = 0.003), whereas this association did not reach significance with the original definition.

Lastly, the relationship between SLEs and state depression is shown in Supplementary Table 5. Patterns were similar to those for trait depression: total SLEs, iSLEs, and dSLEs were all significantly and positively associated with state depression, and no significant differences were found between *β*_iSLEs_ and *β*_dSLEs_. As expected, state depression bore numerically weaker relationships with lifetime SLEs measures than trait depression.

## Discussion

We examined two models, stress-diathesis and stress generation, to explain the links between negative stressful life events (SLEs) and symptoms experienced by participants with schizophrenia spectrum disorders (SSD). Participants with SSD reported a significantly higher number of total SLEs compared to community controls. We separated SLEs into likely independent (iSLEs) and potentially dependent SLEs (dSLEs) and probed different symptom domains. Positive symptom severity was significantly and positively associated with total SLEs; iSLEs and dSLEs showed equal association with symptom severity supporting the stress-diathesis hypothesis. Negative symptom severity was significantly but negatively associated with total SLEs and dSLEs but not iSLEs. This is consistent with the stress generation effect in the negative direction. Depressive symptom severity was significantly and positively associated with total SLEs. The strength of association with dSLEs was not statistically stronger than that with iSLEs, suggesting stress-diathesis but not stress generation effects. This study highlights the importance of a domain-specific approach to characterizing the relationship between life events and major clinical symptoms in schizophrenia. Different underlying processes may govern the relationship between SLEs and symptom severity and call for different intervention strategies.

Participants with SSD reported significantly more total SLEs and dSLEs than community controls with similar age, sex, and racial backgrounds. They were more likely to report suffering from a serious illness, serious injury, or traffic accident; unusual or stressful work or school; and having a hospitalization due to mental illness or substance use problems. Since we did not assess whether these events took place before or after illness onset, these findings cannot be taken as evidence that these events are risk factors for SSD. Events such as hospitalization due to mental illness or substance use problems are likely related to having SSD and underscore that individuals with SSD are vulnerable to having stressful experiences due to their illness. Conversely, individuals with SSD reported fewer divorces or breakup of personal relationships, presumably because they form fewer personal relationships. Lastly, individuals with SSD were more likely to report parental divorce or breakup before they reached age 18, consistent with the literature on adverse childhood experiences as a risk factor for mental illness [47–49].

The total number of SLEs was significantly and positively associated with the severity of positive symptoms in SSD. The beta coefficients for dependent and independent events (*β*_dSLEs_ and *β*_iSLEs_) were both positive. Although only *β*_dSLEs_ reached statistical significance, we did not find statistically significant difference between *β*_dSLEs_ and *β*_iSLEs_. Our failure to replicate the previously reported positive relationship between independent life events and positive symptom severity [19, 23] may be due to problems with how we defined iSLEs. As can be seen in Supplementary Table 4, when not including “divorce or break-up of a relationship” and “lost a primary job, or substantial financial loss”, two events arbitrarily deemed independent if they occurred before age 18, iSLEs had a significant positive relationship with positive symptom severity. Overall, the pattern we observed between iSLEs and positive symptoms appeared most consistent with the stress-diathesis hypothesis (Fig. 1A) and with previous reports that SLEs longitudinally predicted increases in positive symptoms in SSD but not vice versa [19, 20, 23]. That our approach, based on the comparison between *β*_iSLEs_ and *β*_dSLEs_, led to the same conclusion as longitudinal studies provides further support for the validity of the heuristics used in this study.

The total number of SLEs was significantly but negatively associated with the severity of negative symptoms in SSD, however, only *β*_dSLEs_ was significant. This pattern is consistent with the stress generation model in the negative direction (Fig. 1B). Patients who experience more severe negative symptoms are less likely to experience dSLEs. For example, patients with asociality are less likely to pursue personal or romantic relationships and therefore less likely to experience divorce or breakup. Previous studies have examined the effect of independent life events on negative symptoms and reported no significant associations [19, 22, 23]. Another study that did not separate iSLEs and dSLEs reported a bidirectional negative loop between the total number of SLEs and negative symptoms [20]. This included a negative stress-diathesis effect where negative life events over the past 6 months predicted fewer negative symptoms over the past month. This finding is counterintuitive and is perhaps an artifact of the sparse follow-up schedule of the study (the visits were 18–72 months apart). Negative symptom changes could have preceded life events between visits, and only spuriously appeared as responses to life events because negative life events were assessed more retrospectively than negative symptoms (past 6 months vs. past month). This example highlights the importance of corroborating evidence from multiple approaches in investigating the relationship between life events and symptoms.

All SLEs were significantly and positively associated with depressive symptoms. This finding agrees with previous studies which suggested that participants with SSD experience depression following SLEs [20, 22], similar to the effects reported in major depression [15, 50]. However, contrary to our hypothesis, the difference between *β*_dSLEs_ and *β*_iSLEs_ failed to reach significance. Thus, the effects of stress generation of depression in people with SSD remain speculative. To our knowledge, only one previous study tested stress generation of depression in SSD [20]. Using a longitudinal design, they found no evidence that more depressive symptoms were associated with more SLEs over time, which is consistent with our findings. One explanation is that the current analysis lacked statistical power. Alternatively, stress generation effects of depression may not apply to SSD, where depressive symptoms are more responses to illness and life stress than manifestation of internalizing traits [51].

The findings of this study should be interpreted with caution due to several limitations. First, we measured lifetime SLEs retrospectively with a brief checklist of primary life event types filled out by participants. This approach is vulnerable to recall bias and incomprehensiveness. Results may not generalize to event types not covered in this study. Second, we classified iSLEs and dSLEs based on convention in the literature, which is prone to error. Although our results were robust to an alternative classification, future studies should ideally determine independence of SLEs with thorough information obtained from interview. Moreover, ordinal scoring of SLE independence based on interview data [15, 52, 53] may provide stronger statistical power than the dichotomizing approach used in this manuscript. Third, differentiating iSLEs and dSLEs is but one approach to probe the model underlying the event-symptom relationship. Notably, the definition of iSLEs only requires that an event is unlikely influenced by one’s behavior in a loose sense, which does not imply that experiencing the event is fully independent from one’s genetic composition, upbringing, G×E interaction, etc. The relationship between iSLEs and symptoms, when present, can be due to either direct causal effects or shared genetic or environmental pathways – a distinction better made with methods such as Mendelian randomization [54]. We emphasize that findings from this study is best considered in conjunction with evidence from other approaches. Last, the size of correlations between SLEs and symptom domains ranged from weak to moderate (maximum = 0.34). Despite these modest relationships across patients, some patients may be more susceptible to the deleterious effects of SLEs, and some may be more likely to experience more or fewer SLEs because of their symptoms. These are important considerations for case conceptualization and treatment planning.

In conclusion, participants with SSD reported more SLEs than healthy controls, highlighting the need for trauma-informed care in SSD for stressful experiences both before and after illness onset. Separating independent and dependent life events provided insight into the relationship between SLEs and symptoms experienced by participants with SSD, which are specific to symptom domains. The relationship with both positive and depressive symptoms was most consistent with the stress-diathesis model. Negative symptoms were only negatively associated with dSLEs, supporting stress generation effects in the negative direction for negative symptoms. In clinical practice, management of positive and depressive symptoms should include stress reduction and psychoeducation on stress as a risk factor for these symptoms. Following SLEs, providers should closely monitor and provide intervention for positive and depressive symptoms, and depressive symptoms deserved particular attention as they may be overshadowed by positive symptoms or confused with negative symptoms. On the other hand, treatment for negative symptoms may be supplemented with assessment of and coping skills for stress as patients may have higher likelihood of experiencing SLEs as their negative symptoms alleviate. Given the relationship between lifetime SLEs (some happened over 10 years ago) and current psychological wellbeing, prevention of and timely intervention for SLEs may constitute an effective strategy for promoting mental health in individuals with susceptibility to schizophrenia.

### Supplementary information

Supplementary Results

## Data Availability

The minimal dataset that would be necessary to interpret, replicate and build upon the methods or findings reported in the article is available upon request made to the corresponding author.
