# Supplementary material for: Effects of independent versus dependent stressful life events on major symptom domains of schizophrenia
Source: Schizophrenia (Heidelb). 2023 Dec 8;9(1):84. doi: 10.1038/s41537-023-00415-3 (PMC10709301; doi:10.1038/s41537-023-00415-3)
Supplement: Supplementary file 1 — Supplementary Results [file 41537_2023_415_MOESM1_ESM.docx]

**Supplementary Results**

**Factor structure in symptoms**

To explore latent patterns in symptoms, we performed an exploratory factor analysis with 28 item-level measures (4 positive symptom items from the Brief Psychiatric Rating Scale (BPRS), 6 negative symptom subscores from the Brief Negative Symptoms Scale (BNSS), and 18 items on the Maryland Trait and State Depression scale (MTSD)). We specified 3 correlated factors and allowed all items to cross-load onto all factors. The exploratory factor analysis reproduced the factor structure assumed in our original analysis (**Supplementary** **Table 1**), where all MTSD items loaded and only loaded onto Factor 1 (a depressive symptom factor), all BNSS items on to Factor 2 (a negative symptom factor), and all BPRS items onto Factor 3 (a positive symptom factor). Factors 1-3 did not significantly correlate with each other (**Supplementary Table 2**).

**Supplementary Table 1.** Item loadings of exploratory factor analysis.

|  | **Factor1** | | **Factor2** | | **Factor3** | |
| --- | --- | --- | --- | --- | --- | --- |
| **Item** | **loading** | ***p*** | **loading** | ***p*** | **loading** | ***p*** |
| BNSS_alogia | -0.180 | 0.800 | **2.150** | **0.000** | -0.074 | 0.961 |
| BNSS_anhedonia | -0.675 | 0.585 | **4.107** | **0.000** | 0.639 | 0.816 |
| BNSS_asociality | -0.285 | 0.698 | **2.276** | **0.000** | 0.773 | 0.612 |
| BNSS_avolition | -0.107 | 0.894 | **2.700** | **0.000** | 0.845 | 0.637 |
| BNSS_bluntaffect | 0.148 | 0.897 | **3.414** | **0.000** | 0.413 | 0.862 |
| BNSS_distress | 0.177 | 0.532 | **0.844** | **0.000** | 0.170 | 0.780 |
| BPRS_concept_disorg | 0.143 | 0.725 | 0.145 | 0.423 | **0.695** | **0.000** |
| BPRS_grandiosity | 0.062 | 0.686 | 0.000 | 0.998 | **0.681** | **0.000** |
| BPRS_hallucination | -0.246 | 0.439 | 0.046 | 0.824 | **0.906** | **0.004** |
| BPRS_unusual_thought | 0.084 | 0.761 | -0.033 | 0.875 | **1.374** | **0.000** |
| MTSD_1 | **-1.347** | **0.008** | -0.082 | 0.677 | -0.614 | 0.526 |
| MTSD_2 | **-1.296** | **0.008** | 0.057 | 0.762 | -0.556 | 0.543 |
| MTSD_3 | **-0.889** | **0.009** | -0.060 | 0.639 | -0.371 | 0.571 |
| MTSD_4 | **-1.085** | **0.010** | -0.147 | 0.367 | -0.447 | 0.581 |
| MTSD_5 | **-1.062** | **0.015** | -0.031 | 0.843 | -0.367 | 0.632 |
| MTSD_6 | **-1.198** | **0.017** | -0.029 | 0.869 | -0.341 | 0.706 |
| MTSD_7 | **-1.097** | **0.013** | -0.024 | 0.881 | -0.469 | 0.557 |
| MTSD_8 | **-0.886** | **0.010** | -0.093 | 0.467 | -0.396 | 0.542 |
| MTSD_9 | **-1.212** | **0.009** | -0.051 | 0.767 | -0.640 | 0.457 |
| MTSD_10 | **-1.075** | **0.014** | -0.071 | 0.684 | -0.331 | 0.679 |
| MTSD_11 | **-0.908** | **0.016** | -0.002 | 0.988 | -0.273 | 0.692 |
| MTSD_12 | **-1.110** | **0.015** | 0.053 | 0.745 | -0.288 | 0.728 |
| MTSD_13 | **-1.271** | **0.008** | 0.035 | 0.846 | -0.571 | 0.529 |
| MTSD_14 | **-0.926** | **0.013** | -0.085 | 0.554 | -0.421 | 0.538 |
| MTSD_15 | **-0.608** | **0.022** | -0.080 | 0.419 | -0.236 | 0.611 |
| MTSD_16 | **-0.897** | **0.017** | -0.106 | 0.462 | -0.324 | 0.635 |
| MTSD_17 | **-0.759** | **0.021** | -0.189 | 0.128 | -0.322 | 0.589 |
| MTSD_18 | **-1.186** | **0.008** | -0.001 | 0.997 | -0.560 | 0.501 |
| Note: BPRS: Brief Psychiatric Rating Scale. BNSS: Brief Negative Symptoms Scale. MTSD: Maryland Trait and State Depression scale. Bold: significant loadings. | | | | | | |

**Supplementary Table 2.** Factor correlation of exploratory factor analysis

|  | Correlation | Standard Error | *p* |
| --- | --- | --- | --- |
| Factor 1 ~~ |  |  |  |
| Factor 2 | 0.229 | 0.136 | 0.093 |
| Factor 3 | -0.599 | 0.389 | 0.123 |
| Factor 2 ~~ |  |  |  |
| Factor 3 | -0.226 | 0.458 | 0.621 |

**Supplementary Table 3.** Prevalence of SLEs in participants with schizophrenia spectrum disorders and community controls.

|  | **Schizophrenia spectrum disorders**  **(n = 286)** | **Community controls**  **(n = 121)** | **Statistics** |
| --- | --- | --- | --- |
| **SLEs** | **n (%)** | **n (%)** |  |
| 1. *death of a spouse, father, mother, child, sibling, or significant other* | 175 (61.2) | 43 (59.5) | n.s. |
| 1. *suffered from a serious illness, serious injury, or traffic accident* | 171 (59.8) | 48 (39.7) | χ^2^(1) = 13.05^***^ |
| 1. *a significant other suffered from a serious illness or a serious injury* | 98 (34.3) | 43 (35.5) | n.s. |
| 1. *divorce or breakup of a relationship (you or your parents), before 18* | 71 (24.8) | 15 (12.4) | χ^2^(1) = 6.77^**^ |
| *divorce or breakup of a relationship (you or your parents), at or after 18* | 75 (26.2) | 59 (48.8) | χ^2^(1) = 19.83^***^ |
| 1. *unusual, extremely stressful work or school* | 174 (60.8) | 60 (49.6) | χ^2^(1) = 3.96^*^ |
| 1. *experienced violence, a sexual assault, or a robbery* | 98 (34.3) | 43 (35.5) | n.s. |
| 1. *lost a primary job, or substantial financial loss (you or your parents), before 18* | 10 (3.5) | 3 (2.5) | n.s. |
| *lost a primary job, or substantial financial loss (you or your parents), at or after 18* | 122 (42.7) | 53 (43.8) | n.s. |
| 1. *experienced a legal dispute or disputes* | 75 (26.2) | 22 (18.2) | n.s. |
| 1. *had a hospitalization due to mental illness or substance use problems* | 232 (81.1) | 5 (4.1) | χ^2^(1) = 204.04^***^ |
| 1. *other serious life event not listed above* | 59 (20.6) | 16 (13.2) | n.s. |
| *Note*. SLEs: stressful life events. n.s.: not significant. ^*^p < 0.05, ^**^p < 0.01, ^***^p < 0.001 | | | |

**Supplementary Table 4.** Associations between alternatively defined independent and dependent SLEs (excluding events 4) and 7)) and positive and negative symptoms as well as trait depression.

| **Symptoms** | **iSLEs** | | | | **dSLEs** | | | | **β_iSLEs_ – β_dSLEs_** |
| --- | --- | --- | --- | --- | --- | --- | --- | --- | --- |
| *Controlling for age and sex* | | | | | | | | | |
|  | β | *t* | df | p | β | *t* | df | p | p |
| positive | 0.19 | 2.97 | 255 | 0.003^**^ | 0.20 | 3.29 | 255 | 0.001^**^ | 0.86 |
| negative | -0.01 | -0.17 | 254 | 0.87 | -0.21 | -3.47 | 254 | 0.0006^***^ | 0.006^**^ |
| trait depression | 0.23 | 3.71 | 266 | 0.0003^***^ | 0.28 | 4.81 | 266 | 2.5×10^-6***^ | 0.44 |
| *Controlling for age, sex, and CPZ equivalents* | | | | | | | | | |
|  | β | *t* | df | p | β | *t* | df | p | p |
| positive | 0.19 | 2.81 | 197 | 0.005^**^ | 0.13 | 1.99 | 197 | 0.049^*^ | 0.48 |
| negative | -0.05 | -0.68 | 197 | 0.50 | -0.24 | -3.38 | 197 | 0.0009^***^ | 0.029* |
| trait depression | 0.22 | 2.93 | 202 | 0.004^**^ | 0.28 | 3.97 | 202 | 0.0001^***^ | 0.43 |
| *Note*. β: standardized coefficients. SLEs: stressful life events. iSLEs: independent SLEs. dSLEs: dependent SLEs. CPZ: chlorpromazine. ^*^p < 0.05, ^**^p < 0.01, ^***^p<0.001. All p values are two-tailed. | | | | | | | | | |

**Supplementary Table 5.** Association between SLEs and state depression.

| **Covariates** | **SLEs** | | | | **iSLEs** | | | | **dSLEs** | | | | **β_iSLEs_ – β_dSLEs_** |
| --- | --- | --- | --- | --- | --- | --- | --- | --- | --- | --- | --- | --- | --- |
|  | β | *t* | df | p | β | *t* | df | p | β | *t* | df | p | p |
| age, sex | 0.23 | 3.71 | 266 | 0.0002^***^ | 0.14 | 2.21 | 260 | 0.028^*^ | 0.25 | 4.18 | 260 | 4.0×10^-5***^ | 0.12 |
| age, sex, CPZ equivalents | 0.24 | 3.24 | 204 | 0.001^**^ | 0.12 | 1.68 | 201 | 0.095 | 0.29 | 4.06 | 201 | 7.1×10^-5***^ | 0.060 |
| *alternatively defined independent and dependent SLEs (excluding events 4) and 7))* | | | | | | | | | | | | | |
|  | β | *t* | df | p | β | *t* | df | p | β | *t* | df | p | p |
| age, sex | - | - | - | - | 0.19 | 3.01 | 266 | 0.003^**^ | 0.15 | 2.49 | 266 | 0.013^*^ | 0.60 |
| age, sex, CPZ equivalents | - | - | - | - | 0.19 | 2.56 | 204 | 0.011^*^ | 0.17 | 2.37 | 204 | 0.019^*^ | 0.86 |
| *Note.* β: standardized coefficients. SLEs: stressful life events. iSLEs: independent SLEs. dSLEs: dependent SLEs. CPZ: chlorpromazine. ^*^p < 0.05, ^**^p < 0.01, ^***^p<0.001. All p values are two-tailed. | | | | | | | | | | | | | |
